# Uncovering translation roadblocks during the development of a synthetic tRNA

**DOI:** 10.1093/nar/gkac576

**Published:** 2022-07-27

**Authors:** Arjun Prabhakar, Natalie Krahn, Jingji Zhang, Oscar Vargas-Rodriguez, Miri Krupkin, Ziao Fu, Francisco J Acosta-Reyes, Xueliang Ge, Junhong Choi, Ana Crnković, Måns Ehrenberg, Elisabetta Viani Puglisi, Dieter Söll, Joseph Puglisi

**Affiliations:** Department of Structural Biology, Stanford University, Stanford, CA 94305-5126, USA; Program in Biophysics, Stanford University, Stanford, CA 94305-5126, USA; Department of Molecular Biophysics and Biochemistry, Yale University, New Haven, CT 06511, USA; Department of Structural Biology, Stanford University, Stanford, CA 94305-5126, USA; Department of Molecular Biophysics and Biochemistry, Yale University, New Haven, CT 06511, USA; Department of Structural Biology, Stanford University, Stanford, CA 94305-5126, USA; Department of Biochemistry and Molecular Biophysics, Columbia University, New York, NY 10032, USA; Department of Biochemistry and Molecular Biophysics, Columbia University, New York, NY 10032, USA; Department of Cell and Molecular Biology, Uppsala University, Uppsala 751 24, Sweden; Department of Structural Biology, Stanford University, Stanford, CA 94305-5126, USA; Department of Molecular Biophysics and Biochemistry, Yale University, New Haven, CT 06511, USA; Department of Cell and Molecular Biology, Uppsala University, Uppsala 751 24, Sweden; Department of Structural Biology, Stanford University, Stanford, CA 94305-5126, USA; Department of Molecular Biophysics and Biochemistry, Yale University, New Haven, CT 06511, USA; Department of Chemistry, Yale University, New Haven, CT 06511, USA; Department of Structural Biology, Stanford University, Stanford, CA 94305-5126, USA

## Abstract

Ribosomes are remarkable in their malleability to accept diverse aminoacyl-tRNA substrates from both the same organism and other organisms or domains of life. This is a critical feature of the ribosome that allows the use of orthogonal translation systems for genetic code expansion. Optimization of these orthogonal translation systems generally involves focusing on the compatibility of the tRNA, aminoacyl-tRNA synthetase, and a non-canonical amino acid with each other. As we expand the diversity of tRNAs used to include non-canonical structures, the question arises as to the tRNA suitability on the ribosome. Specifically, we investigated the ribosomal translation of allo-tRNA^UTu1^, a uniquely shaped (9/3) tRNA exploited for site-specific selenocysteine insertion, using single-molecule fluorescence. With this technique we identified ribosomal disassembly occurring from translocation of allo-tRNA^UTu1^ from the A to the P site. Using cryo-EM to capture the tRNA on the ribosome, we pinpointed a distinct tertiary interaction preventing fluid translocation. Through a single nucleotide mutation, we disrupted this tertiary interaction and relieved the translation roadblock. With the continued diversification of genetic code expansion, our work highlights a targeted approach to optimize translation by distinct tRNAs as they move through the ribosome.

## Graphical Abstract

**Graphical Abstract:**
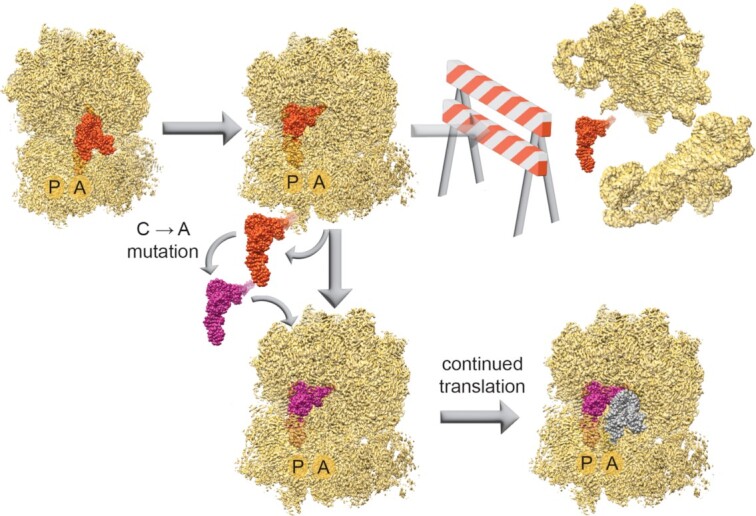
A single nucleotide mutation in tRNA^UTu1^ removes the translation roadblock causing ribosome disassembly and permits continued translation.

## INTRODUCTION

**Figure 1.**
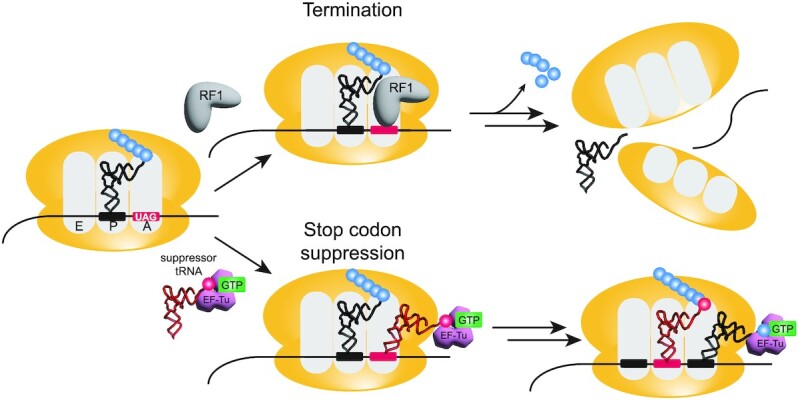
Genetic recoding at stop codons competes with release factors. In the presence of a UAG stop codon, one of two situations can occur: termination or suppression. Termination of translation occurs when release factor 1 (RF1) binds, releasing the polypeptide chain and invoking separation of the ribosomal subunits from the mRNA. Alternatively, in the presence of a suppressor tRNA, the UAG codon can be suppressed, inserting an amino acid and allowing continuation of translation.

Protein synthesis is a highly conserved process performed by the ribosome. Aminoacyl-tRNA synthetases (aaRSs) acylate the 3′-terminal CCA sequence with the cognate amino acid to produce aminoacyl-tRNAs (aa-tRNAs) that are carried to the ribosome by EF-Tu. With 46 aa-tRNA substrates in *Escherichia coli* (1,2), the ribosome has evolved to accommodate and discriminate between all tRNAs for efficient translation (3). Given that extensive contacts form between the ribosome and aa-tRNA, this is a remarkable feature that is only possible due to the malleability of the ribosome and conserved tRNA structure (4). The ribosome has specifically evolved for translation of aa-tRNAs from the same organism, but also has the capability to perform heterologous translation, accepting aa-tRNAs from other organisms or domains of life. The orthogonality of some tRNA:aaRS pairs between domains of life allows for translation systems from other organisms to be used for genetic recoding and insertion of non-canonical amino acids (ncAAs) (5,6). Genetic recoding typically is performed at traditional stop codons (e.g. UAG, UAA or UGA), to introduce a ncAA. The efficiency and specificity of genetic recoding relies first on the speed and accuracy of aa-tRNA synthesis, and second on the decoding and translocation function of the tRNA as it moves through the ribosome (Figure 1).

**Figure 2.**
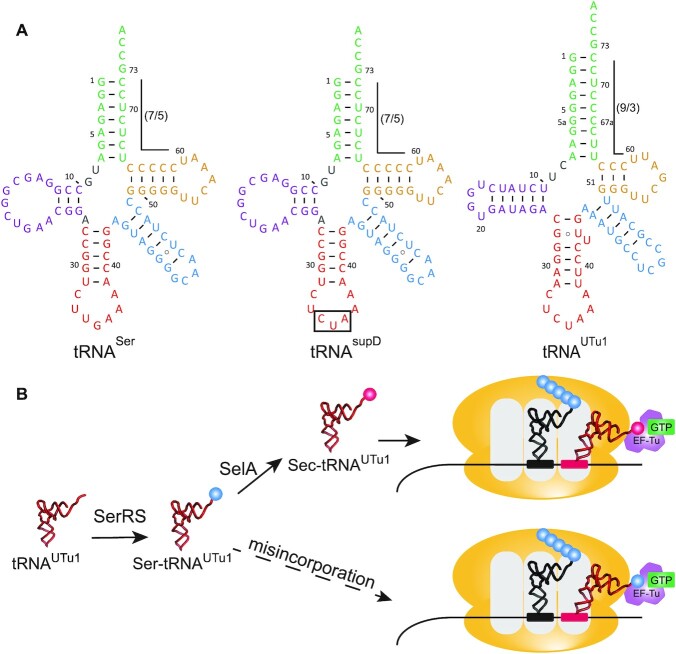
EF-Tu driven selenocysteine incorporation. (**A**) Cloverleaf structures of *E. coli* tRNA^Ser^, tRNA^supD^ (the amber suppressor of tRNA^Ser^) and tRNA^UTu1^. Residues are colored as follows: acceptor arm (green), D-arm (purple), anticodon arm (red), variable arm (blue), and T-arm (gold). (**B**) Translation schematic of EF-Tu driven selenocysteine incorporation with tRNA^UTu1^. tRNA^UTu1^ is first serylated by seryl-tRNA synthetase (SerRS) to generate Ser-tRNA^UTu1^. Selenocysteine synthase (SelA) converts the serine to selenocysteine to generate Sec-tRNA^UTu1^. Aminoacylated tRNA^UTu1^ (including Ser-tRNA^UTu1^ which is misincorporated) are substrates for EF-Tu, inserting at the UAG codon for continued translation.

Substantial engineering efforts have focused on the former; *Methanosarcina* PylRS (7) and *Methanocaldococcus jannaschii* TyrRS with their cognate tRNAs have become well-established orthogonal translation systems. These tRNA synthetases and their variants facilitated the successful incorporation of several hundred different ncAAs programmed by UAG or UAA (8). An alternate natural translation system with potential for genetic recoding is the insertion of selenocysteine (Sec) programmed by UGA. The high reactivity and unique chemical properties of Sec have attracted the attention of synthetic biologists for various applications. However, due to the requirement of Sec-specific translation components (mRNA hairpin recognition element and elongation factor SelB), genetic code expansion applications for Sec have been limited. Consequently, research focused on engineering a simpler translation pathway that could be applied for site-specific insertion of Sec in any protein. This strategy involves engineering tRNA^Sec^ to be recognized by the natural elongation factor (EF-Tu), following the translation pathway of the other 20 canonical amino acids. Initial attempts to create a hybrid tRNA between tRNA^Sec^ and tRNA^Ser^ for recognition by EF-Tu resulted in low protein yields and minimal Sec insertion (9–12). Therefore, an alternate approach searched for other tRNAs that resembled tRNA^Sec^ and are capable of EF-Tu recognition. A search of metagenomic sequences revealed uniquely structured tRNAs (allo-tRNAs) containing recognition elements that resembled tRNA^Sec^ but with a 12 bp acceptor domain (acceptor and T-stem combined) for proper recognition by EF-Tu (13). Specifically, tRNA^UTu1^ was found to be a serine isoacceptor with a 9/3 (acceptor/T-stem bp) structure that allowed EF-Tu driven Sec incorporation (Figure 2).

These allo-tRNAs are non-native substrates for the *E. coli* ribosome, therefore their translation efficiency is unknown. For seamless translation, tRNAs must be quickly accepted into the ribosomal A site and efficiently translocated to the P site (14,15). Disturbing these processes can lead to stalling and disassembly of the translating ribosome, reducing protein synthesis. This has been observed with quadruplet tRNAs, which are poor substrates for the ribosome due to the four-base anticodon-codon interaction (16–18). This roadblock has been tackled separately by mutating either the tRNA in the anticodon loop to promote the four-base anticodon-codon interaction (19,20) or engineering an orthogonal ribosome (Ribo-Q) efficient at decoding quadruplet codons (21).

Here, we investigated the suitability of an allo-tRNA, tRNA^UTu1^, for translation by *E. coli* ribosomes, and demonstrate the importance of global tRNA structure for efficient translation. tRNA^UTu1^ is used for site-specific insertion of Sec by harnessing EF-Tu to avoid constraints on natural Sec translation. Using single-molecule FRET (Förster resonance energy transfer) we visualized movement of tRNA^UTu1^ through the ribosome and compared it to natural tRNAs (tRNA^Ser^ and tRNA^supD^). From this, we were able to establish the roadblocks associated with tRNA^UTu1^ translation through the ribosome. Implementing cryo-EM, we visualized tRNA^UTu1^ in complex with the *E. coli* ribosome to pinpoint the structural feature in tRNA^UTu1^ hindering translation. With this information, we engineered a single-base mutation in tRNA^UTu1^ that removed the rigidity of the variable arm, enhancing translational processivity on the ribosome. This was verified through *in vivo* and *in vitro* assays and led to an increase in recombinant protein production. Our results demonstrate the importance of ensuring that synthetic tRNAs used for genetic code expansion are suitable substrates for ribosomal translation and show the power of combined dynamics and structural analyses for improved tRNA engineering.

## MATERIALS AND METHODS

### Single-molecule translation assay

Details on preparation of reagents for the single-molecule assay are described in the Supplemental Information. Using a previously described protocol, the 30S pre-initiation complex (30S PIC) was prepared from Cy3B-labeled 30S subunit, ribosomal protein S1, initiation factor IF2, fMet-tRNA^fMet^, biotinylated mRNA and 4 mM GTP. Prior to immobilizing the 30S PIC, we prepared the SMRT Cell v3 from Pacific Biosciences (Menlo Park), a ZMW chip, with Neutravidin. The 30S PIC was then diluted to 15 nM and loaded into the SMRT cell (22).

Ternary complexes (TCs) of Phe(Cy5)-tRNA^Phe^ and Ser-tRNAs were formed with EF-Tu (GTP) using a previously described protocol (23). After formation of TCs, a 2X delivery mix was created with 100–400 nM Cy5-TC^Phe^ and 400 nM-2 μM TC^Ser^ along with 200 nM BHQ-2-50S, 100 nM EF-G, 4 mM GTP, 2.5 mM Trolox and the oxygen scavenging system (PCA and PCD). Before starting an experiment, the delivery mix was diluted into polymix mixture in the SMRT Cell and loaded into a modified PacBio RSII sequencer (24). At the start of the experiment, the instrument illuminated the SMRT cell with a green laser and then transferred 20 μl of a delivery mixture onto the cell surface at *t* = 10 s. All experiments were performed at 20°C, and data was collected for 6 min.

### Cryo-EM

Details on preparation of grids and data processing are described in the Supplemental Information. Data was collected on a Titan Krios transmission electron microscope (Thermo Fisher Scientific) equipped with a K3 Summit direct electron detection camera (Gatan) in counting mode. Micrographs were accrued at a calibrated pixel size of 1.037 Å and with a nominal defocus range of –1 to –2 μm. Each micrograph consisted of 40 frames collected over a two second exposure at a dose rate of roughly 20 electrons per pixel per second for a total dose of roughly 40 electrons/Å^2^. The micrographs were acquired as dose-fractionated image stacks.

### sfGFP readthrough assay

sfGFP readthrough was performed as previously described (9) using plasmids pB_sfGFP (UKGE or UGTT), pSecUAG (with tRNA^supD^, tRNA^UTu1^ or tRNA^UTu1A^) and *B-95*.ΔAΔ*fabR E. coli* cells (25). Each experiment was performed with a minimum of four biological replicates and background subtracted (no IPTG).

### GPx1 protein production

Expression and purification of human GPx1 was performed as previously described (9,11). Briefly, pET-GPx1(49UAG) and pSecUAG (with tRNA^supD^, tRNA^UTu1^ or tRNA^UTu1A^) were transformed into *B-95*.ΔAΔ*fabR E. coli* cells (25). Cultures were grown at 37°C and immediately induced with 0.1% arabinose for tRNA expression with 10 μM sodium selenite for Sec formation. Cells were grown until OD_600_ 1.2, at which point the temperature was lowered to 20°C, and protein expression was induced for 20 hrs. Purified protein samples were sent to Bioinformatics Solutions, Inc (Canada) for intact mass spectrometry to quantify Sec incorporation. Spectra were analysed using Thermo BioPharma Finder™ 4.1 (ThermoFisher Scientific).

## RESULTS

### tRNA^UTu1^ induces low translational processivity

**Figure 3.**
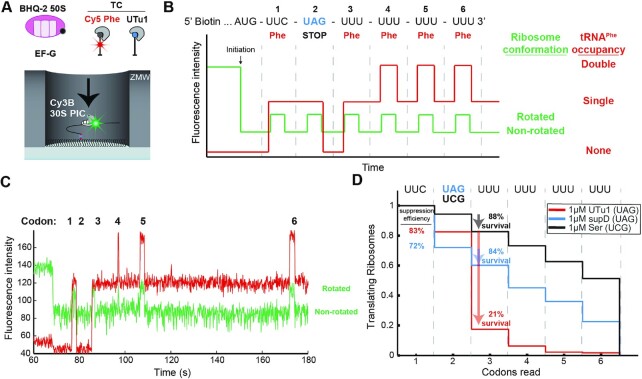
Single-molecule characterization of low translation processivity induced by tRNA^UTu1^. (**A**) In all single-molecule experiments, 30S preinitiation complexes (PIC) containing Cy3B-30S, fMet-tRNA^fMet^, and IF2 is immobilized on the surface of the ZMW wells through biotinylated mRNAs. The reaction is started by delivery of BHQ-2-50S, Cy5-TC^Phe^, EF-G, and one of the serine-charged suppressor tRNA TCs. (**B**) Expected sequence of fluorescence signals starting with quenching of Cy3B (green) that signals 50S subunit joining (initiation) and six cycles of changes in Cy3B intensity that signal intersubunit rotations during elongation. First and last four elongation cycles are correlated with Cy5 intensity changes that signal Cy5-tRNA^Phe^ binding. (**C**) Representative single-molecule trace shows successful ribosome translation of all six codons after UAG stop codon suppression by tRNA^supD^. (**D**) Codon survival plot of the fraction of translating ribosomes as a function of number of codons translated. In the presence of 1μM tRNA^UTu1^ (red, *n* = 223) ribosome survival (percent of codon 2 ribosomes that survive codon 3) is drastically decreased compared to the same amount of tRNA^supD^ (blue, *n* = 186) or tRNA^Ser^ (black, *n* = 265).

tRNA^UTu1^ was identified in a metagenomic search for use in site-specific, EF-Tu driven, Sec insertion (13,26). Since tRNA^UTu1^ is not a canonical substrate for *E. coli* translation we decided to initially explore the dynamics of tRNA^UTu1^ stop-codon suppression within the *E. coli* ribosome. This was accomplished using a single-molecule fluorescence assay and zero-mode waveguide (ZMW)-based instrumentation (24). We observed the progression of translation in real time at single-codon resolution by tracking the ribosome conformational changes underlying elongation. The ribosomal intersubunit rotational movements during elongation were monitored using a small (30S) subunit site-specifically labelled with Cy3B and a large (50S) subunit labelled with BHQ-2 (a non-fluorescent quencher), allowing for FRET between the two dyes (27). In addition to ribosomes labeled with FRET pairs, Cy5-labeled tRNA^Phe^ was used to track the tRNA occupancy on the ribosome during translation (Figure 3A). We applied this approach to monitor decoding of the UAG stop codon by serylated tRNA^UTu1^ (Ser-tRNA^UTu1^) and compared it to a canonical serylated tRNA capable of UAG suppression (Ser-tRNA^supD^).

This approach allowed us to monitor translation elongation and stop-codon suppression with different tRNAs. The initial low Cy3B signal in the single-molecule trace is a result of the BHQ-2-50S subunit quenching Cy3B on the 30S subunit to form the 70S non-rotated state during translation initiation (Figure 3B). Subsequent decoding of the first Phe codon prompts the arrival of Cy5-TC^Phe^, signaled by a burst in Cy5 fluorescence. Successful peptide bond formation is visualized by medium Cy3B intensity from a FRET transition due to the ribosome intersubunit rotation into the rotated state. This successful decoding event is then followed by EF-G-driven translocation of Cy5-tRNA^Phe^ from the A site to the P site, placing the UAG stop codon in the A site and driving intersubunit rotation back to the non-rotated state (low Cy3B intensity). Each Cy5-tRNA^Phe^ occupancy lasts for two cycles of elongation as it translocates from A to P and then P to E sites. Therefore, elongation of consecutive Phe codons downstream of the stop codon results in cycles of double- and single-occupancy states, making the signal for translation downstream of stop codon unequivocal (Figure 3B). Following the changes in fluorescence intensity, we can track the processivity of translation (Figure 3C).

Translation with Ser-tRNA^UTu1^ yielded a decoding efficiency at the UAG stop codon (codon 2) of 83%. Similar suppression efficiency was measured when translating in the presence of Ser-tRNA^supD^ (72%) (Figure 3D). tRNA^supD^ (identical in sequence to *E. coli* tRNA^Ser^ but with an anticodon to decode UAG) provides us with information on the suppression efficiency of a natural substrate for the ribosome. Since decoding of UAG is known to be slower than a sense codon, we directly compared the two suppressor tRNAs (tRNA^UTu1^ to tRNA^supD^) but also measured tRNA^Ser^ as a control using single-molecule fluorescence. The decoding time is calculated from the dwell time of the non-rotated state prior to ribosome rotation at which point the tRNA is successfully incorporated and peptidyl transfer occurs (Supplementary Figure S1A). Decoding of tRNA^UTu1^ showed a similar distribution of decoding times at UAG as tRNA^supD^ with means of ∼7 s at 1 μM TC. This is approximately twice as long as the decoding time of tRNA^Ser^ that decodes the sense codon UCG (Supplementary Figure S1B). Due to limitations in the *in vitro* single-molecule setup (detection times for signals and tRNA/factor concentrations), decoding times are not equivalent to those observed during cellular translation.

With suppression efficiency and decoding time of tRNA^UTu1^ matching the natural suppressor tRNA^supD^, we next investigated what occurs after translocation of the suppressor tRNA from the A to the P site. After UAG suppression by tRNA^UTu1^, we found a significant drop in the ribosome processivity at the third codon. At low concentrations (50 nM) of Cy5-TC^Phe^, only 21% of ribosomes continued translating following tRNA^UTu1^ incorporation, compared to 84% of ribosomes that continued translating after tRNA^supD^ insertion (Figure 3D). The low ribosome survival after tRNA^UTu1^ incorporation was predominantly due to ribosome disassembly pathways. This was manifested as either a 50S subunit dissociation from the immobilized 30S complex or a 70S ribosome dissociation from the immobilized mRNA signaled by the dequenching of Cy3B signal (Supplementary Figure S2A) or disappearance of Cy3B signal (Supplementary Figure S2B), respectively. Of the ribosomes that incorporated tRNA^UTu1^, 9% underwent one of these two possible ribosome disassembly events before the subsequent translocation step and 64% underwent disassembly after translocation (Supplementary Figure S2C). In contrast, the frequency of this post-translocation disassembly was only 1% for ribosomes that incorporated tRNA^supD^ (Supplementary Figure S2D). We tested to see whether this pathway competes with the subsequent arrival of the Cy5-TC^Phe^ to codon 3 by increasing the concentration of the Cy5-TC^Phe^ that was delivered from 50 to 200 nM. This resulted in a 2.5-fold reduction of the frequency of post-translocation ribosome disassembly (Supplementary Figure S3A) and increased the overall processivity on codon 3 to 72% (Supplementary Figure S3B). These results suggest an intrinsic ribosome instability introduced upon successful incorporation of tRNA^UTu1^. This instability is predominantly present after the next translocation that places tRNA^UTu1^ in the P site. With a fast measured ribosome disassembly time of 9 ± 1 s at room temperature, this intrinsic instability poses a problem to the overall translational yield after stop codon suppression.

### The variable arm of tRNA^UTu1^ forms a unique tertiary interaction

**Figure 4.**
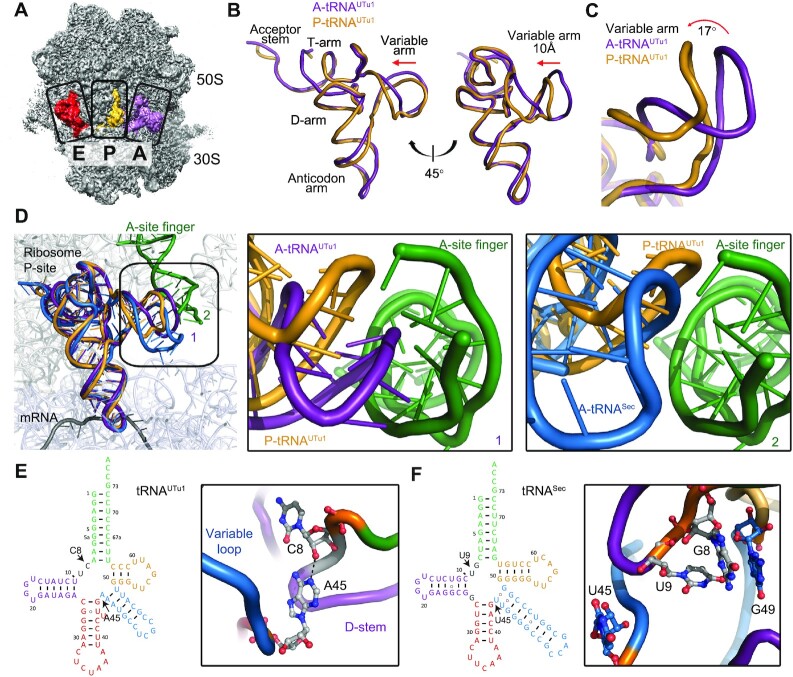
Structural studies of tRNA^UTu1^ on the ribosome. (**A**) Structure of the ribosome, highlighting tRNAs in the A, P and E sites. (**B**) Overlay of A/A (purple, PDB:7UR5) and P/P (orange, PDB:7UR5) state tRNA^UTu1^ highlights the shift in the variable arm position. (**C**) Zoom in on the variable arm shows P/P tRNA^UTu1^ (orange) to rotate 17° upwards from A/A tRNA^UTu1^ (purple). (**D**) Overlay of A/A (purple) and P/P (orange) state tRNA^UTu1^ with A/A state tRNA^Sec^ (blue, PDB:5LZE) in the context of the ribosome P site. A clash is observed with the A/A state tRNA^UTu1^ and the A-site finger of the ribosome. A zoom in on the variable arm with respect to the A-site finger shows the shift from the A/A to the P/P state for tRNA^UTu1^ and compares it with the A/A state of tRNA^Sec^ (blue). (**E**) Cloverleaf structure of tRNA^UTu1^ highlights the position of the residues of interest, C8 and A45. A zoom in on the structure of those residues shows the formation of a hydrogen bond. (**F**) Cloverleaf structure of tRNA^Sec^ highlights the corresponding residues of interest from tRNA^UTu1^. A zoom in on the structure of those residues (PDB:5LZE) shows that there is no base pair present. Instead, the corresponding residues point outwards, away from the tRNA core.

tRNA^UTu1^ processivity when bound in the ribosomal P site is significantly decreased (Figure 3D) as shown by the high frequency of post-translocation ribosome disassembly (Supplementary Figure S2C); we supposed that the novel structure of tRNA^UTu1^ may be responsible for this lack of processivity and translation complex destabilization. To visualize structures and orientations of tRNA^UTu1^ in the ribosome, we used single-particle cryo-EM on samples of tRNA^UTu1^ in complex with the *E. coli* 70S ribosome. Due to the high frequency of ribosome disassembly, cryo-EM was advantageous to sort out the lowly-populated ribosome–tRNA^UTu1^ complex from vacant ribosomes. The complex was prepared by programming tRNA^UTu1^ to bind in all ribosomal sites using an mRNA containing three tandem UAG codons (28). After cryo-EM image processing, we observed a class of particles with a non-rotated 70S ribosome state bound to triple tRNA^UTu1^ in the A/A, P/P and E/E states (PDB:7UR5, Figure 4A and Supplementary Figure S4). The global resolution of the triple tRNA^UTu1^ and ribosome complex is 2.6 Å, allowing atomic-level modeling (Supplementary Figure S4).

tRNAs form a conserved cloverleaf secondary structure with four helices: the acceptor stem, the D-arm, the anticodon arm, and the T-arm. The clover leaf folds into a three-dimensional L-shaped structure through coaxial stacking of the acceptor stem/T-stem and D-stem/anticodon stem mediated by tertiary interactions from the variable loop to the D-stem and D-loop to T-loop pairing. The length of the variable loop between the anticodon stem and the T-arm, divides the tRNAs into two classes. Class I tRNAs have a variable loop of 4–5 nucleotides, such as tRNA^Phe^, and class II tRNAs have a much longer variable loop of 10–24 nucleotides, such as tRNA^Sec^ (29). tRNA^UTu1^ belongs to class II, in which the variable loop is long enough to form a fifth helix. The global structural features of the tRNA^UTu1^ acceptor stem and the anticodon stem of tRNA^UTu1^ are similar to previously published tRNA structures in the A site, including tRNA^Phe^ (PDB: 4V6F) and tRNA^Sec^ (PDB: 5LZE) (Supplementary Figure S5A–I). The distance and angles between the acceptor stem and anticodon stem are conserved, allowing tRNA^UTu1^ to reach into both the peptidyl transferase center and the decoding center, essential for tRNA function. The longer acceptor stem of 9 bp leads to a shift of 9.6 Å between the central loop linker G8 of tRNA^Sec^ and C8 of tRNA^UTu1^. The compensation of a shorter T-arm of 3 bp, leads to a shift of 6.5 Å between G49 of tRNA^Sec^ and U49 of tRNA^UTu1^ (Supplementary Figure S5J–K).

Despite the presence of a tetraloop in the D-loop, the tertiary structure of tRNA^UTu1^ is stabilized by canonical T-loop to D-loop pairing of G18:U55 and G19:C56. In addition, tRNA^UTu1^ forms a base pair between U16:U59 which is similar to C16:C59 of tRNA^Sec^ but not observed in tRNA^Phe^ between U16 and U59 (Supplementary Figure S5D–F). Furthermore, the D-arm of tRNA^UTu1^ consists of only Watson-Crick base pairs, whereas in both tRNA^Sec^ and tRNA^Phe^, nucleotides from the central loop interact in the major groove of the D-arm helix to form base triples (Supplementary Figure S5G–I). Overall, the structural features of tRNA^UTu1^ in the ribosomal A site led to an 8.7 Å shift of the variable loop as compared to tRNA^Sec^ (Supplementary Figure S5J–K).

When tRNA^UTu1^ is bound in the P site, both the acceptor stem and the anticodon stem conformations are conserved, allowing them to reach into both the peptidyl transferase center and the decoding center. The variable loop of tRNA^UTu1^ is shifted by 10 Å compared with its conformation in the A site. This movement creates a 17° rotation of the variable loop upwards towards the ribosome (Figure 4B, C) to avoid a clash with the A-site finger (ASF), a functional attenuator for translocation (30). The central loop and the T-loop to D-loop interaction networks in P site tRNA^UTu1^ are similar to A site tRNA^UTu1^ (Supplementary Figure S6). There are to date no published structures of tRNA with a long variable loop at the P site for comparison. Superimposing the A/A tRNA^Sec^ into the P site does not result in a clash of the variable loop with the ASF (31) (Figure 4D). These results imply that while A/A tRNA^Sec^ can theoretically fit into the P site as is, translocation of tRNA^UTu1^ requires modulation of the variable loop orientation. The additional constraint on tRNA^UTu1^ variable loop orientation imposed by the ribosomal scaffold could raise the energetic barrier for tRNA^UTu1^ translocation, and steric clash could lead to ribosome disassembly, as observed by single-molecule FRET (Supplementary Figure S2C). If this hypothesis is true, mutating tRNA^UTu1^ to allow for higher flexibility or alternative orientation of the variable arm would lower the energetic barrier to P site accommodation and reduce ribosome splitting.

Modulation of the flexibility or orientation of the variable arm could be achieved by redesigning the base of the variable arm, at the central loop that forms the four-way junction in the tRNA. The central loop of tRNA^UTu1^ does not interact with any of the stems, but A45 base paring with C8 was identified as a key stabilizing element of the central loop (Figure 4E). The A45:C8 base pair is sandwiched between A46:U48 and U9 through stacking interactions (Supplementary Figure S5G). Although uncommon, A:C mismatch base-pair exists in both RNA (e.g. initiator tRNA) (32,33) and DNA structures (34). In contrast, tRNA^Phe^ (PDB: 4V6F) and tRNA^Sec^ (PDB:5LZE) central loop nucleotides interact with the D-arm forming base triples and do not form strong interactions with each other. Specifically, the corresponding residues in tRNA^Sec^ (U45 and U9) point in opposite directions with G49 of the variable arm protruding into the D-arm to stabilize the tertiary structure (31) (Figure 4F). We hypothesized that disrupting the A45:C8 interaction might destabilize the central loop, which could lead to higher flexibility of the variable loop, resulting in lower rates of ribosome splitting and improved processivity of ribosomes after suppression.

### A single base mutation in tRNA^UTu1^ increases processivity and protein yield

**Figure 5.**
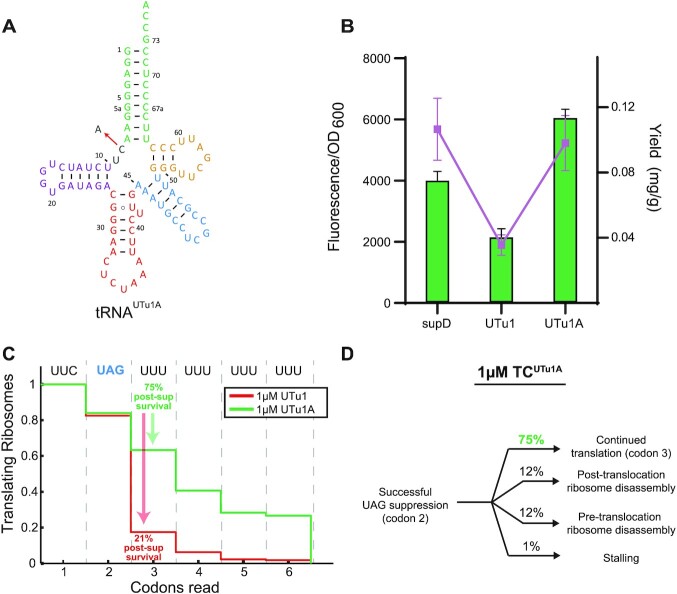
Single nucleotide mutation enhances translation processivity. (**A**) Cloverleaf structure of tRNA^UTu1A^ showing the single nucleotide C to A change from tRNA^UTu1^ by the red arrow. (**B**) sfGFP fluorescence readthrough assay (green bars) and GPx1 protein yields (purple dots) demonstrate the increased processivity of tRNA^UTu1A^ compared to tRNA^UTu1^. Fluorescence reads are shown as an average of four biological replicates (± standard deviation) and protein yields are an average of two biological replicates (± standard deviation). (**C**) Codon survival curve plotting the fraction of translating ribosomes as a function of number of codons translated highlights the post-suppression survival (percent of codon 2 ribosomes that survive codon 3) advantage in the presence of 1 μM tRNA^UTu1A^ (green, *n* = 300) over 1 μM tRNA^UTu1^ (red, *n* = 223). (**D**) Pathway map of ribosomes that successfully suppressed a UAG stop codon in the presence of 1 μM tRNA^UTu1A^.

To test the above hypothesis, we chose to mutate C8 since the connecting regions between the acceptor arm and D-arm are not known to interact with any of the components involved in Sec translation. We made a C8A mutation to reduce the likelihood that a base pair would form with A45 (tRNA^UTu1A^, Figure 5A). To test the effect of this mutation *in vivo*, we designed a fluorescence readthrough assay that mimics the low processivity state of tRNA^UTu1^ (Supplementary Figure S7A). With the observation that codon processivity was increased upon the addition of 200 nM Cy5-TC^Phe^ compared to 50nM (Figure 3D, Supplementary Figure S3), we devised a system in which the availability of the subsequent tRNA was low, such as in a ribosomal stalling event. Following a study that investigated ribosomal stalling motifs (35), we chose to mutate the three amino acids following the amber codon (position 2 of sfGFP) to one of the top 20 stalling motifs (protein sequence GTT) (Supplementary Figure S7B). To confirm that this strategy generated an *in vivo* assay that reflected our *in vitro* data, we tested tRNA^UTu1^ and tRNA^supD^ for UAG readthrough of sfGFP with the N-terminal protein sequence UKGE (non-ribosomal stalling motif) and UGTT (ribosomal stalling motif). No significant difference was observed between the tRNAs with UKGE, however, a significant decrease in fluorescence was found for tRNA^UTu1^ compared to tRNA^supD^ with UGTT (Supplementary Figure S5C, D). These results demonstrate that we have tuned the traditional sfGFP readthrough assay (sfGFP_UGTT) to probe tRNA variants for increased P site processivity.

Testing tRNA^UTu1A^ with our sfGFP_UGTT assay, we observed a 3-fold increase in fluorescence compared to tRNA^UTu1^ (Figure 5B). This result was further verified when we used tRNA^UTu1^ and tRNA^UTu1A^ for expression of human GPx1 (a natural selenoprotein with a GTT stalling motif after the amber codon). We found that tRNA^UTu1A^ expressed roughly three times more protein (0.036 ± 0.004 mg/g) compared to tRNA^UTu1^ (0.010 ± 0.001 mg/g) (Figure 5B) with no significant change in the amount of selenocysteine (∼10%) (Supplementary Figure S8). Moreover, the protein yield achieved by tRNA^UTu1A^ was comparable to the natural suppressor, tRNA^supD^.

Real-time single-molecule FRET experiments confirmed that tRNA^UTu1A^ processivity on the ribosome P site increased significantly (75%) relative to tRNA^UTu1^ (21%) (Figure 5C and D), equivalent to what was observed for tRNA^supD^ (Figure 3D). Results from intact mass spectrometry of purified GPx1 suggest that the effect of low processivity found for tRNA^UTu1^ is transferred throughout the length of the protein. ESI-MS found that tRNA^UTu1^ had a significant amount of truncated protein (51%) which was drastically decreased for tRNA^UTu1A^ (15%) (Supplementary Figure S8A, B).

### Disruption of tertiary interaction in tRNA^UTu1^ removes rigidity of its variable arm

To investigate how the C8A mutation boosted tRNA^UTu1A^ processivity on the ribosome P site, we generated a cryo-EM structure using the same strategy described above (i.e. capturing A and P site tRNA^UTu1A^ on the ribosome). Data processing and particle sorting resulted with two maps, each with either A site tRNA^UTu1A^ or P site tRNA^UTu1A^ alone. A site tRNA^UTu1A^ has a similar density to tRNA^UTu1^ and was modelled with a similar global fold (PDB:7URI, Supplementary Figure S9). The P site tRNA^UTu1A^ anticodon stem, acceptor arm, and elbow region density are well defined and similar to tRNA^UTu1^. The stabilized central loop in tRNA^UTu1A^ is modified by C8A mutation through forming the A8 and A45 interaction while the variable loop of P site tRNA^UTu1A^ has poor cryo-EM density and could not be traced with high confidence (PDB:7URM). Highly flexible RNA chains are often poorly defined in cryo-EM, which in turn can point to higher flexibility of the variable loop of tRNA^UTu1A^ in accordance with our single-molecule FRET data presented above (Figure 5C). Moreover, molecular dynamic simulations of each tRNA in solution highlights the increased fluctuations of the variable arm as well as the D- and T-arms (Supplementary Figure S10). Thus, the structural and functional data support the need for flexibility in the suppressor tRNA conformation to accommodate the different ribosomal sites during translation. By engineering tRNA^UTu1^ to eliminate the stabilizing interactions, we were able to increase its suppression efficiency.

## DISCUSSION

Here, we combined functional, dynamic and structural methods to understand translation of a synthetic tRNA (tRNA^UTu1^) by the *E. coli* ribosome. Together, our approach allowed us to identify a structural element in tRNA^UTu1^ that hinders processivity in the ribosome P site. A single nucleotide mutation (C8A) removed the translational roadblock, increasing protein expression to match that of a natural suppressor tRNA (tRNA^supD^) (Figure 5B).

Efficient translation requires tRNAs to be optimal substrates for the ribosome with smooth transitions between the A, P and E sites. Details about this journey through the ribosome are emerging with the focus on natural tRNA substrates (36,37). Molecular dynamic simulations have found that the ASF plays a significant role in the transition of tRNAs from the A to the P site, interacting with the T-loop of the tRNA (38). Further interactions were observed with type II tRNAs containing a long variable arm (such as tRNA^Ser^, tRNA^Leu^ and tRNA^Tyr^ in *E. coli*). These additional variable arm contacts are suggested to be the driving force which move the ASF for the tRNA to translocate between the A and P site (39). However, little is known about the details of the variable arm and ASF interactions, making it difficult to engineer a synthetic tRNA^Sec^ that can efficiently translocate through the ribosome.

Movement of the ASF is only possible due to its inherent flexibility; an essential feature for tRNAs with long variable arms to be accommodated in the A/P hybrid state on the ribosome (39). As can be imagined, this flexibility must be modulated and the variable arm should move cooperatively with the ASF to reduce strain on the system. With tRNA^UTu1^, a synthetic tRNA species to *E. coli*, we observed that its long variable arm must move significantly to avoid a steric clash with the ASF for translocation from the A to the P site (Figure 4C). This uncooperative behavior likely strains the ribosome and results in disassembly and poor translational processivity (Figure 3D). We found a unique C8:A45 base pair in the core of tRNA^UTu1^, which may promote the stabilization of the variable arm and prevent flexibility needed for cooperative movement with the ASF.

The choice for disrupting the C8:A45 base pair was 2-fold. (i) C8 was chosen for mutagenesis over A45 to minimize any effect that a change of sequence in the variable arm would have on serylation. Although the chance of this would be small considering that SerRS specifically recognizes the structure of the long variable arm, rather than the sequence, this was avoided (40). Moreover, the similar suppression efficiencies of tRNA^UTu1^ compared to tRNA^UTu1A^ (Figure 5C) suggest that serylation was not affected. (ii) The choice of base for substitution at position 8 was also carefully chosen. Uracil, being the predicted Watson–Crick pair, was an obvious exclusion while guanine is also known to commonly form a mispairs (41). It followed that mutation to an adenine would have the highest likelihood of preventing an interaction with A45. The structure of tRNA^UTu1A^ revealed this disrupted interaction without altering the position of the variable arm in the A site. Instead, the absence of the C8:A45 mismatch base pair in the core of tRNA^UTu1A^ created flexibility in the variable arm such that it could move cooperatively with the ASF in transition from the A to the P site (Supplementary Figure S9). This movement is likely favorable for translation and prevents ribosome disassembly.

Our discovery emphasizes one reason why it is difficult to engineer a translation system that is efficient in site-specific Sec incorporation. The initial barrier of SECIS (selenocysteine insertion sequence)-dependent translation with SelB was overcome by rewiring translation to be EF-Tu compatible. This breakthrough allowed Sec to be site-specifically incorporated into any protein (10–12). Efforts to improve the system led to the discovery of tRNA^UTu1^ (13), which significantly increased the protein yield compared to previous systems (9). However, as our work shows there is room for improving tRNA^UTu1^ as well as other synthetic tRNAs proposed for Sec insertion (26). Specifically, we highlight that the variable arm, which is necessary for serylation, can pose a problem in translation through the ribosome. Capturing the tRNA structure in the ribosome provided a map to visualize and target the tRNA regions necessary to solve this translational roadblock.

This knowledge can apply to other synthetic tRNAs that are used for translation in *E. coli*. tRNA^Pyl^ and *M. jannaschii* tRNA^Tyr^, which are both highly used for genetic code expansion applications, have small variable arms, similar in size to tRNA^Phe^. The movement required of the ASF is minimal for the tRNA to reach the A/P hybrid state, interacting only with the T-loop of the tRNA (38,39). However, quadruplet tRNAs are being engineered from tRNAs with and without variable arms (19,42–44) Therefore, consideration as to the suitability of their variable arms in the *E. coli* ribosome is imperative to ensure efficient A to P site translocation. Moving forward with designing synthetic tRNAs, care should be taken when considering the tRNA scaffold to maximize protein yield and prevent translational roadblocks.

## DATA AVAILABILITY

Atomic coordinates and structure factors for the reported cryo-EM structures have been deposited with the Protein Data Bank under accession numbers 7UR5, 7URI and 7URM and the Electron Microscopy Data Bank under accession numbers EMD-26705, EMD-26713 and EMD-26714.

## Supplementary Material

gkac576_Supplemental_File

## Notes

Present address: Arjun Prabhakar, Pacific Biosciences, Inc., Menlo Park, CA 94025, USA.

Present address: Ana Crnković, Laboratory for Molecular Biology and Nanobiotechnology, National Institute of Chemistry, Hajdrihova 19, Ljubljana, Slovenia.
