# Supplementary material for: Uncovering translation roadblocks during the development of a synthetic tRNA
Source: Nucleic Acids Res. 2022 Jul 27;50(18):10201–11. doi: 10.1093/nar/gkac576 (PMC9561287; doi:10.1093/nar/gkac576)
Supplement: gkac576_Supplemental_File [file gkac576_supplemental_file.pdf]

SUPPLEMENTARY DATA

Uncovering translation roadblocks during the development of a synthetic tRNA

Arjun Prabhakar[1,2†], Natalie Krahn[3,†], Jingji Zhang[1,†], Oscar Vargas-Rodriguez[3], Miri Krupkin[1], Ziao Fu[4], Francisco J Acosta-Reyes[4], Xueliang Ge[5], Junhong Choi[1], Ana Crnković[3], Måns Ehrenberg[5], Elisabetta Viani Puglisi[1], Dieter Söll[3,6,*], Joseph Puglisi[1,*]

[1] Department of Structural Biology, Stanford University, Stanford, CA, 94305-5126, USA
[2] Program in Biophysics, Stanford University, Stanford, CA 94305-5126, USA.
[3] Department of Molecular Biophysics and Biochemistry, Yale University, New Haven, CT, 06511, USA
[4] Department of Biochemistry and Molecular Biophysics, Columbia University, New York, NY 10032, USA
[5] Department of Cell and Molecular Biology, Uppsala University, Uppsala 751 24, Sweden
[6] Department of Chemistry, Yale University, 225 Prospect St, New Haven, CT, 06511, USA

[*]To whom correspondence should be addressed. Tel: 1-650-498-4397; Email: puglisi@stanford.edu
Correspondence may also be addressed to Dieter Söll; Email: dieter.soll@yale.edu

[†]These authors contributed equally to the work

Present Addresses:
Arjun Prabhakar, Pacific Biosciences, Inc., Menlo Park, CA 94025, USA.

Ana Crnković, Laboratory for Molecular Biology and Nanobiotechnology, National Institute of Chemistry, Hajdrihova 19, Ljubljana, Slovenia

This PDF includes:

Supplementary methods

Figures S1-S10

SUPPLEMENTARY METHODS

Preparation of plasmids

tRNA genes for UTu1, supD, Ser, and UTu1A were cloned into pUC18 using NEBuilder® HiFi DNA assembly (NEB).

Escherichia coli codon optimized sfGFP with C-terminal His_6-tag and entire lacI system (including the T7 promoter and terminator) was amplified from a pET-15b vector and moved into a pBAD plasmid where it replaced the araBAD promoter (pB_sfGFP) (1). Site-directed mutagenesis using *Pfu*Ultra II Fusion HS DNA Polymerase (Agilent) was done to introduce a TAG codon at position 2 (UKGE_sfGFP). pB_sfGFP was amplified with primers that encoded amino acids GTT at positions 3-5 (UGTT_sfGFP) followed by NEBuilder® HiFi DNA assembly (NEB) to reassemble the plasmid.

Construction of the Sec expression system plasmid containing $tRNA^{UTu1}$ was previously reported (pSecUAG) (2) and modified for spectinomycin resistance (1). $tRNA^{supD}$ was inserted into pSecUAG as previously described (1). Site directed mutagenesis using *Pfu*Ultra II Fusion HS DNA Polymerase (Agilent) was performed to mutate a single nucleotide (C8A) on $tRNA^{UTu1}$ to generate $tRNA^{UTu1A}$.

Construction of pET-GPx1(49UAG) has been previously described (2).

Preparation of reagents for single-molecule translation assay

Mutant *E. coli* 30S and 50S ribosomal subunits containing hairpin loop extensions in 16S rRNA helix 44 and 23S rRNA helix 101 were constructed and purified as described before (3,4). IF2, EF-Tu, EF-G, EF-Ts, and ribosomal protein S1 from *E. coli* were purified from overexpressing strains as previously described (4). fMet-$tRNA^{fMet}$ was prepared from aminoacylating native $tRNA^{fMet}$ according to published protocols (5,6). Phe-(Cy5)$tRNA^{Phe}$ was prepared by first fluorescently labeling native $tRNA^{Phe}$, then purifying and aminoacylating Cy5-$tRNA^{Phe}$ according to published protocols (5,6). The MF-UAG and MF-UCG mRNA, chemically synthesized by Dharmacon, contains a 5′-biotin followed by a 5′-UTR and Shine-Dalgarno sequence derived from gene 32 of the T4 phage upstream of the AUG start codon. There are four Phe codons (UUU) downstream of the UAG stop codon or UCG Ser codon for MF-UAG and MF-UCG, respectively.

E. coli cells from the ASKA library (7) harboring the EcSerRS.pCA24N plasmid were used for overexpression of *E. coli* seryl-tRNA synthetase (SerRS). Cells were grown at 37°C to an OD_{600} of 0.6 before induced for protein overexpression with 1 mM IPTG for 4 hrs. Cells were harvested by centrifugation and resuspended in 50 mM sodium phosphate buffer (pH 8) and 300 mM NaCl. Cells were lysed with lysozyme and by sonication. *E. coli* SerRS was purified using TALON® Metal Affinity Resin (TAKARA) following the manufacturer's protocol. The protein was eluted with an imidazole gradient and stored in buffer containing 25 mM sodium phosphate (pH 8), 150 mM NaCl, and 40% glycerol at -20°C. Protein concentrations were calculated using the Bradford assay (BioRad).

DNA template for *in vitro* transcription was PCR-amplified from the corresponding pUC18 plasmid using a M13F primer together with reverse primers complementary to the 3'-end of each tRNA. Transcription reac-

tions were carried out with ~10 μg of DNA, 10 mM Tris-HCl (pH 8), 20 mM $MgCl_2$, 2 μg/mL yeast pyrophosphatase, 1 mM spermidine, 0.01% Triton X-100, 5 μg/mL BSA, 5 mM DTT, 4 mM each nucleotide tri-phosphate (ATP, GTP, UTP, and CTP) together with T7 RNA polymerase for 7 hrs at 37°C. tRNAs were separated on a 12% urea-PAGE and extracted with 500 mM ammonium acetate and 1 mM EDTA (pH 8). T7 RNA polymerase was prepared following a previously described protocol (8).

The *in vitro* transcribed $tRNA^{UTu1}$, $tRNA^{supD}$, $tRNA^{Ser}$, and $tRNA^{UTu1A}$ were aminoacylated with Ser using purified *E. coli* SerRS (described above). Each aminoacylation reaction contained 4 μM SerRS and 8 μM tRNA in buffer containing 50 mM HEPES (pH 7.3), 4 mM ATP, 10 mM $MgCl_2$, 0.1 mg/mL BSA, 1 mM DTT, and 0.5 mM L-Ser. After incubation of the aminoacylation reaction at 37°C for 45 mins the reaction was quenched with 300 mM sodium acetate (pH 4.5). Standard acid phenol-chloroform extraction and ethanol were used to isolate the serylated tRNAs. Ser-tRNAs were run through a Sephadex G25 spin column (GE Healthcare) to eliminate any ATP. The tRNA concentration was determined using a NanoDrop (Thermo Scientific).

All single-molecule experiments were conducted in a Tris-based polymix buffer consisting of 50 mM Tris-acetate (pH 7.5), 100 mM KCl, 5 mM ammonium acetate, 0.5 mM calcium acetate, 5 mM magnesium acetate, 0.5 mM EDTA, 5 mM putrescine-HCl, and 1 mM spermidine. Prior to the single-molecule experiments, the purified 30S and 50S ribosomal subunits (final concentration 1 μM) were mixed in a 1:1 ratio with the fluorescent dye-labeled DNA oligonucleotides complementary to the mutant ribosome hairpin extensions (3,4) at 37 °C for 10 mins and then at 30 °C for 20 mins in the Tris-based polymix buffer system. The 30S subunit was labeled with 5'-Cy3B-labeled DNA and 50S subunit was labeled with 3'-BHQ-2-labeled DNA.

RSII instrumentation and data analysis

Single-molecule intersubunit FRET and tRNA occupancy experiments were conducted using a commercial PacBio RSII sequencer. The sequencer was modified to allow the collection of single-molecule fluorescence intensities from individual ZMW wells about 130 nm in diameter in four different dye channels corresponding to Cy3, Cy3.5, Cy5, and Cy5.5 fluorescence. The RSII sequencer has two lasers for dye excitation at 532 nm and 632 nm. In all experiments, data was collected at 10 frames per second (100 ms exposure time) for 6 mins using energy flux settings of the green laser at 0.60 mW/mm^2 and red laser at 0.10 mW/mm^2.

Data analyses for all experiments were conducted with MATLAB (MathWorks) scripts written in-house (9). Briefly, fluorescence traces from each ZMW were automatically selected based on fluorescence intensity, fluorescence lifetime, and the changes in intensity. Filtered traces exhibiting intersubunit FRET (Cy3B-BHQ-2) and $tRNA^{Phe}$ binding at the first codon (Cy5) were then manually curated for further data analysis. The FRET states were assigned as previously described (10) based on a hidden Markov model-based approach and visually corrected. Lifetimes measured of the different FRET states were collected across a large sample of ZMW traces (n>100) to provide a representative distribution from which a mean and standard error was calculated on MATLAB.

Cryo-EM specimen preparation

Specimens were composed of vitrified samples occupying UltrAuFoil R 2/2, 300-mesh holey Au/Au grids (Quantifoil Micro Tools). The surfaces of the grids were rendered hydrophilic by glow-discharging using H_2 and O_2 for 25 s at 10 W with a Solarus 950 plasma cleaner system (Gatan). For vitrification, 3 µL of the sample was applied to each grid, blotted for 3 s at a blot force of 3 inside a Vitrobot Mark IV (Thermo Fisher Scientific), and plunge-froze in liquid propane:ethane mixture (63:37, v/v) cooled with liquid nitrogen. The temperature of the specimen was kept 20°C.

Cryo-EM data processing

The beam-induced motion of the sample and the instability of the stage due to thermal drift was corrected using MotionCor2 (11). The contrast transfer function (CTF) of each micrograph was estimated using CTFFIND4 (12). Imaged particles were picked using the Autopicker algorithm included in the RELION software (13).

For $tRNA^{UTu1}$ in complex with the ribosome (Supplementary Figure S4), 2,668,059 particles were picked from 11,492 micrographs. 2D classification of the four-times binned particles were used to separate ribosome-like particles from ice-like and/or debris-like particles picked by the Autopicker algorithm. 1,605,175 particles were saved for 3D classification. Of those, 1,207,471 particles were selected from high-resolution 3D classes for focused 3D classification. Focused 3D classification was performed on re-extracted particles without binning. 631,624 particles with $tRNA^{UTu1}$ density were selected for auto-refinement.

For $tRNA^{UTu1A}$ in complex with the ribosome (Supplementary Figure S7), 10,850,093 particles were picked from 23,738 micrographs. 8,024,280 good particles were saved for 3D classification after 2D classification. Two major classes, non-rotated 70S (682,020 particles) and rotated 70S (179,559 particles) were saved for forced 3D classification based on the tRNA densities. For non-rotated classes, a major class (424,828 particles) containing A site tRNA and a minor class (23,609) containing P site tRNA were obtained from masked 3D classification. For rotated classes, two classes were found. One class (88,852 particles) contained E site tRNA and another class (67,556 particles) contained both A/P and P/E tRNAs.

Un-binned particles from these classes were subjected to auto-refinement. The final density map was sharpened by applying a negative B-factor estimated by automated procedures. Local resolution variations were estimated using ResMap (14) and visualized with UCSF Chimera (15).

Model building and refinement

Models of the *E. coli* 70S ribosome (5WE4) were docked into the maps using UCSF Chimera (15). All models were manually adjusted, and then de-novo built for the missing residues in Coot (16) followed by Phenix (17) and Refmac (18) refinement. All figures showing electron densities and atomic models were created using USCF Chimera (15) and PyMol Molecular Graphics Systems.

Molecular dynamic simulations

The starting P site cryo-EM tRNA structures of both $tRNA^{UTu1}$ (PDB:7UR5) and $tRNA^{UTu1A}$ (PDB:7URM) were taken out of the ribosome for simulation in solution. Nine Mg ions were added to the tRNA structures by superimposing the tRNA backbone with $tRNA^{Phe}$ (PDB:1EHZ) and extracting the Mg ion's position from that structure. Each tRNA was solvated in a dodecahedron-shaped box (edge length approximately nm) with TIP3P water molecules (19) and neutralized with 0.15M NaCl. The nucleic acid structure and ions were described using the CHARMM36 force field (20). All simulations were performed using the GROMACS (version 2021.4) package (21,22). To maintain constant temperature and pressure, a V-rescale and C-rescale coupling were applied, respectively. The tRNAs were equilibrated to a temperature of 300 K with a coupling time of 0.1 ps and pressure of 1 bar with a coupling time of 1.0 ps. The isothermal compressibility was 4.5 x 10^{-5} bar^{-1}. A leap-frog integrator with an integration time step of 0.002 fs was used. The H-bonds were constrained using P-LINCS algorithm (23) with a short-range electrostatic and van der Waals cutoff of 1.2 nm. Three independent replicas were performed for each molecular system. The three trajectories were combined. From this an average structure was generated and root mean squared fluctuations (RMSF) were calculated for each base.

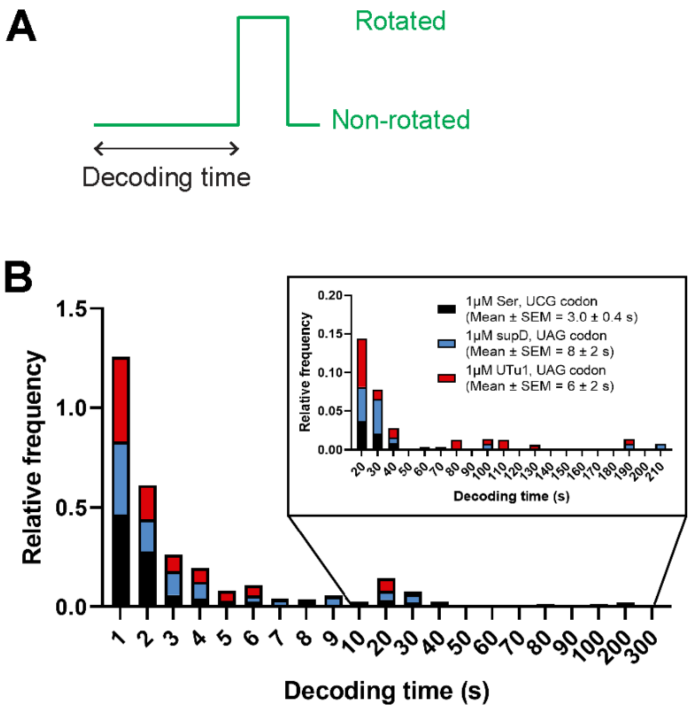

Supplementary Figure S1. Decoding times of $tRNA^{UTu1}$, $tRNA^{supD}$, and $tRNA^{Ser}$. (**A**) Decoding time of a codon is defined as the dwell time of the non-rotated ribosome state before the intersubunit rotation for the elongation cycle of the corresponding codon. (**B**) Histogram of the distribution of codon 2 decoding times in the presence of 1 µM $tRNA^{Ser}$ (n = 250), $tRNA^{supD}$ (n = 134) or $tRNA^{UTu1}$ (n = 184). The mean and S.E.M. of the distributions are reported in parentheses.

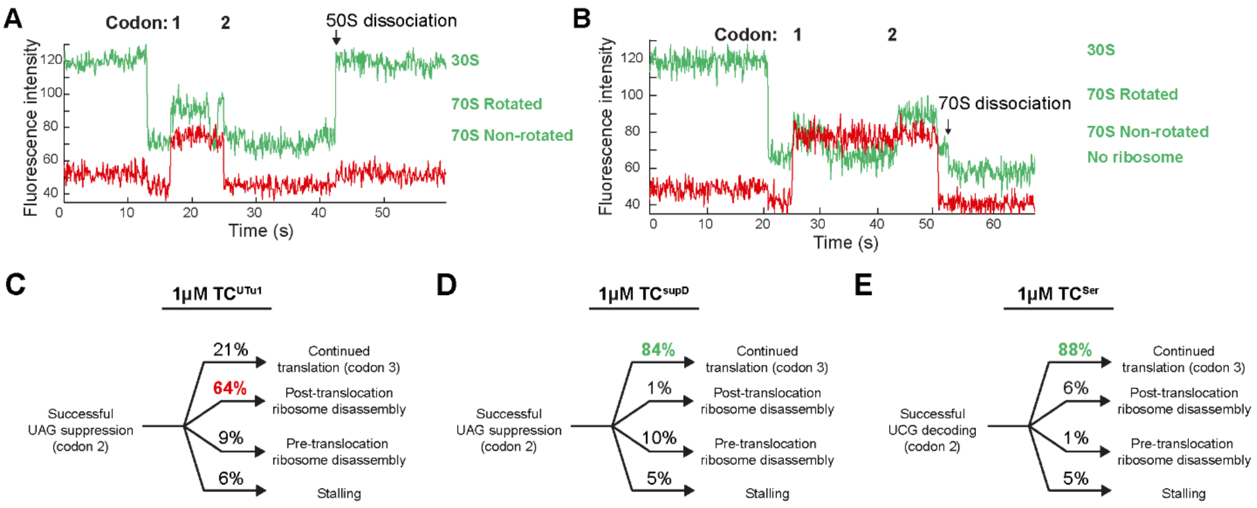

Supplementary Figure S2. Ribosome disassembly pathways after successful tRNA^{UTu1} suppression. (**A**) Representative single-molecule trace of a post-translocation 50S subunit dissociation event that followed successful UAG suppression by tRNA^{UTu1}. (**B**) Representative single-molecule trace of a post-translocation 70S subunit dissociation event that followed successful UAG suppression by tRNA^{UTu1}. (**C**) Pathway map of ribosomes that successfully suppressed a UAG stop codon in the presence of 1 μM tRNA^{UTu1}. (**D**) Pathway map of ribosomes that successfully suppressed a UAG stop codon in the presence of 1 μM tRNA^{supD}. (**E**) Pathway map of ribosomes that successfully decoded a UCG codon in the presence of 1 μM tRNA^{Ser}.

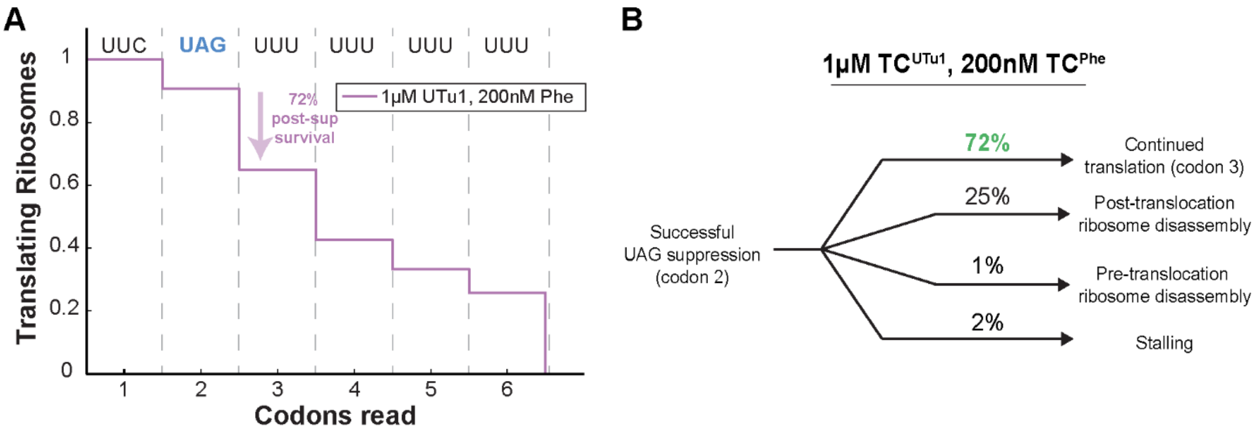

Supplementary Figure S3. Processivity of tRNA^{UTu1} increases with excess Phe-tRNA^{Phe}. (**A**) Codon survival curve plotting the fraction of translating ribosomes (n = 225) as a function of the number of codons translated. (**B**) Pathway map of ribosomes that successfully suppressed a UAG stop codon in the presence of 1 μM tRNA^{UTu1}. Ribosome survival increases for tRNA^{UTu1} with an increase to 200 nM of the Phe ternary complex (TC).

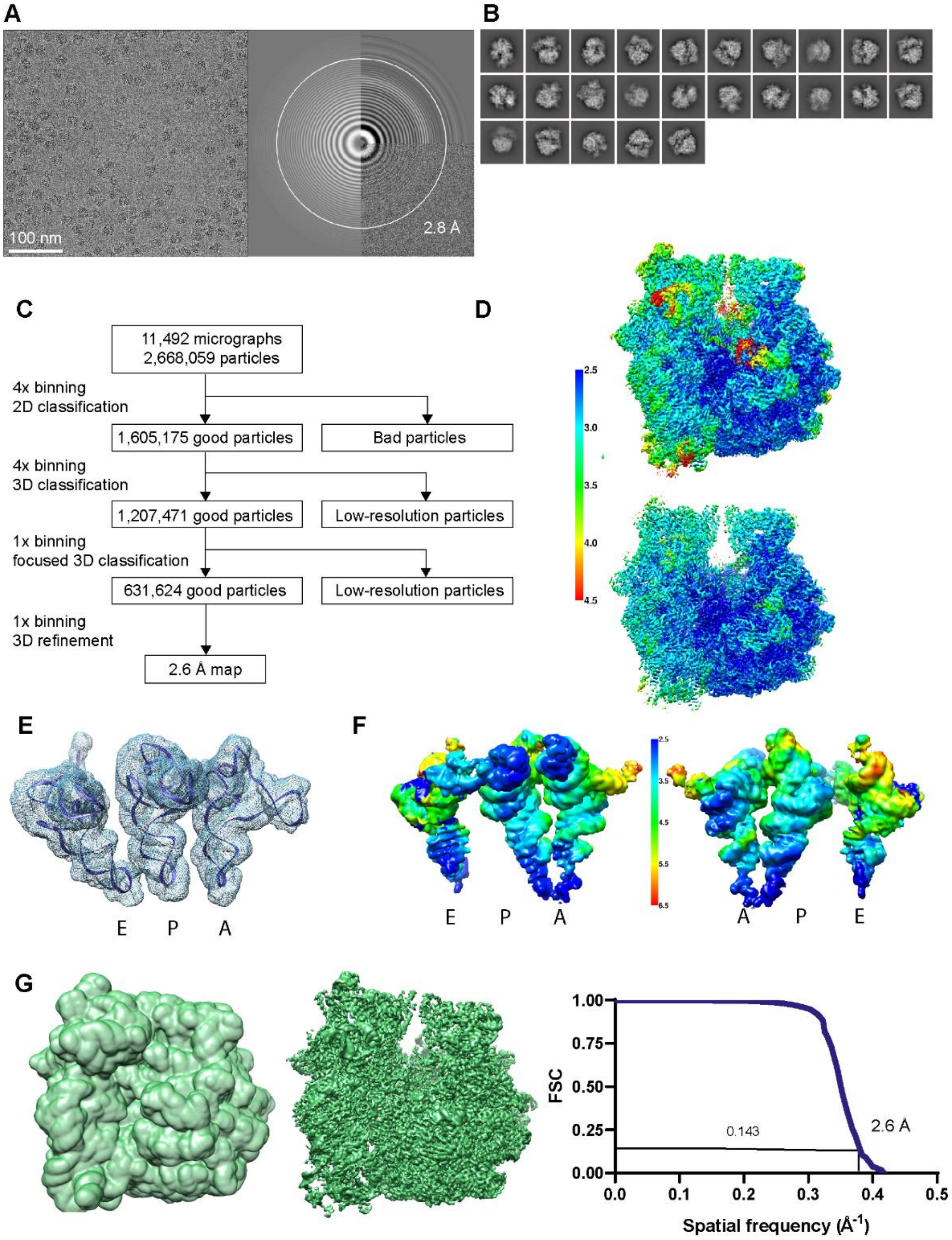

Supplementary Figure S4. Cryo-EM data processing of tRNA^{UTu1} in complex with the ribosome. (**A**) Representative micrograph collected with a Titan Krios transmission electron microscope and corresponding power spectrum. (**B**) Representative 2D class averages from reference-free 2D classification. (**C**) Particle classification and structural refinement procedures used. (**D**) Local resolution estimation of the cryo-EM density map of the ribosome. The density map is displayed in surface representation and colored according to the local resolution (see color bar). (**E**) tRNA^{UTu1} in the E, P, and A site of the ribosome. (**F**) Local resolution estimation of the cryo-EM density map of tRNA^{UTu1} in the E, P, and A site of the ribosome. The density map is displayed in surface representation and colored according to the local resolution (see color bar). (**G**) The mask used for resolution estimation (left) including all components of the reconstruction. FSC curve for cryo-EM reconstruction (right).

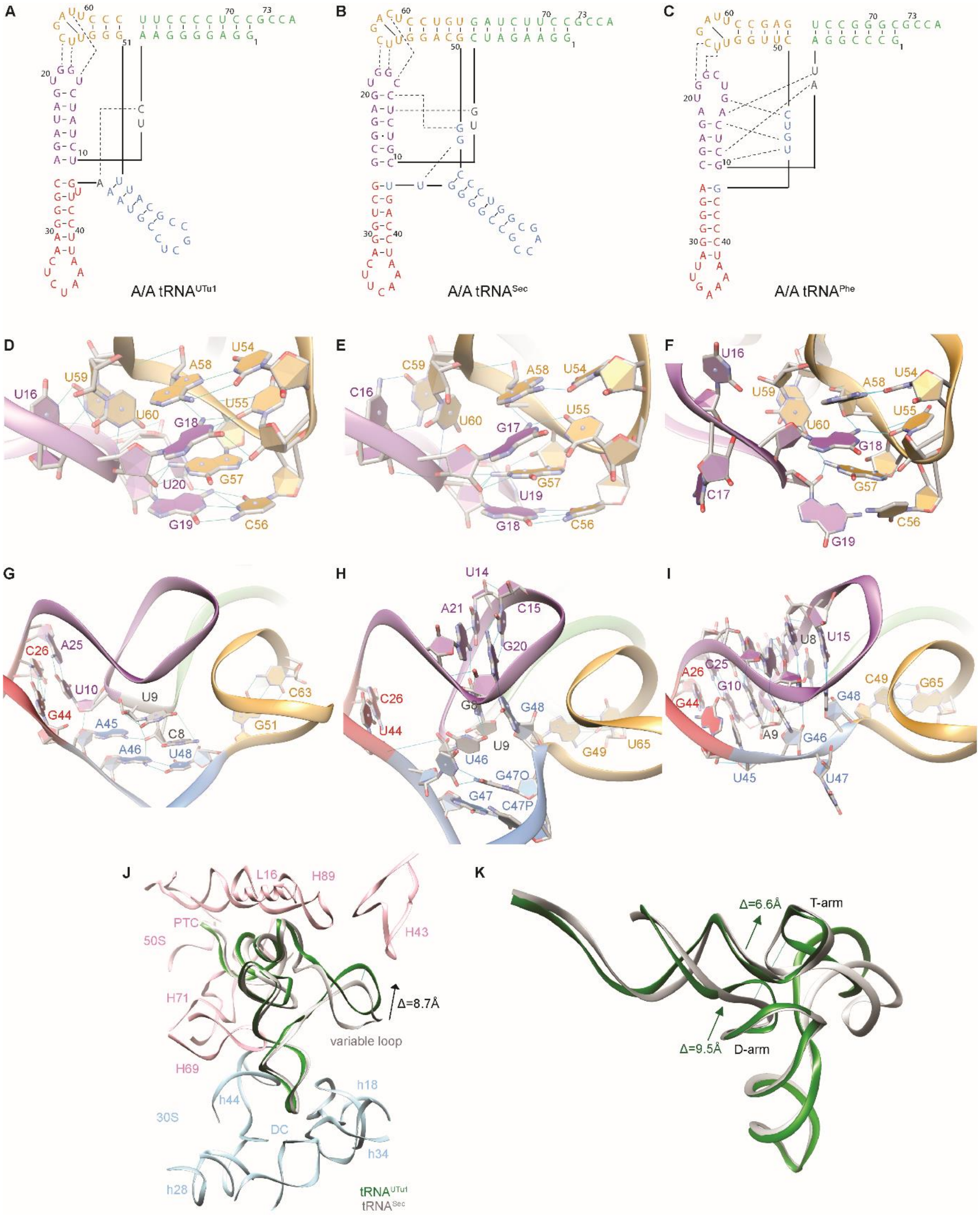

Supplementary Figure S5. Conformation of A/A tRNA^{UTu1}. Simplified network of base pairing for (**A**) tRNA^{UTu1} (PDB:7UR5), (**B**) tRNA^{Sec} (PDB:5LZE), and (**C**) tRNA^{Phe} (PDB:4V6F) in the A site of the ribosome. (**D-F**) Tertiary interactions shown in the T-loop-D-loop, and (**G-I**) central loop interactions shown with the D-stem. (**J-K**) Comparison of tRNA^{UTu1} and tRNA^{Sec} conformations in A site highlight the differences in the variable loop position. H-bonds are shown in teal.

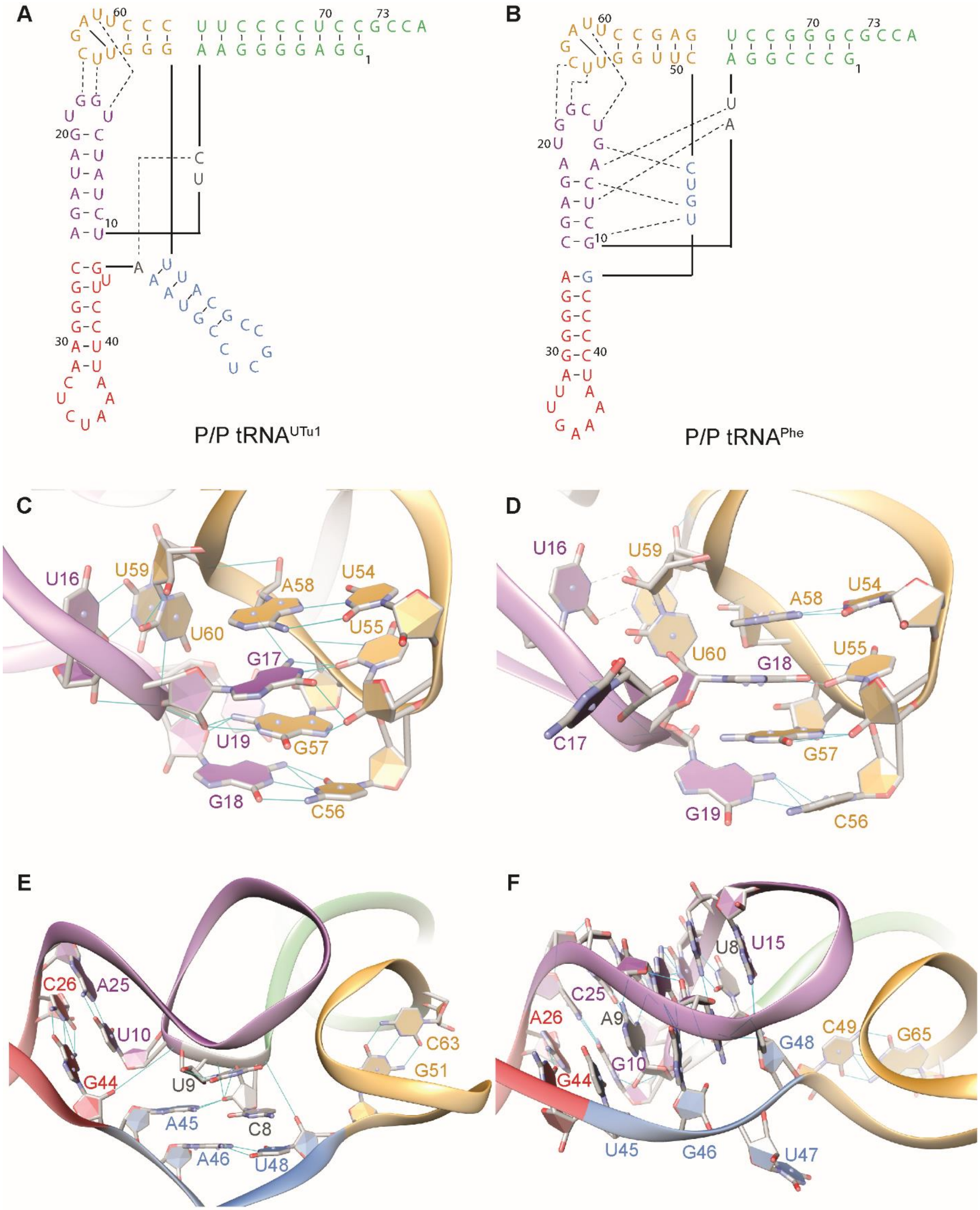

Supplementary Figure S6. Conformation of P/P tRNA^{UTu1}. Simplified network of base pairing for (**A**) tRNA^{UTu1} (PDB:7UR5) and (**B**) tRNA^{Phe} (PDB:4V6F) in the P site of the ribosome. (**C-D**) Tertiary interactions shown in the T-loop-D-loop, and (**E-F**) central loop interactions shown with the D-stem. Notice that there is a double conformation for U9 of P/P tRNA^{UTu1}. H-bonds are shown in teal.

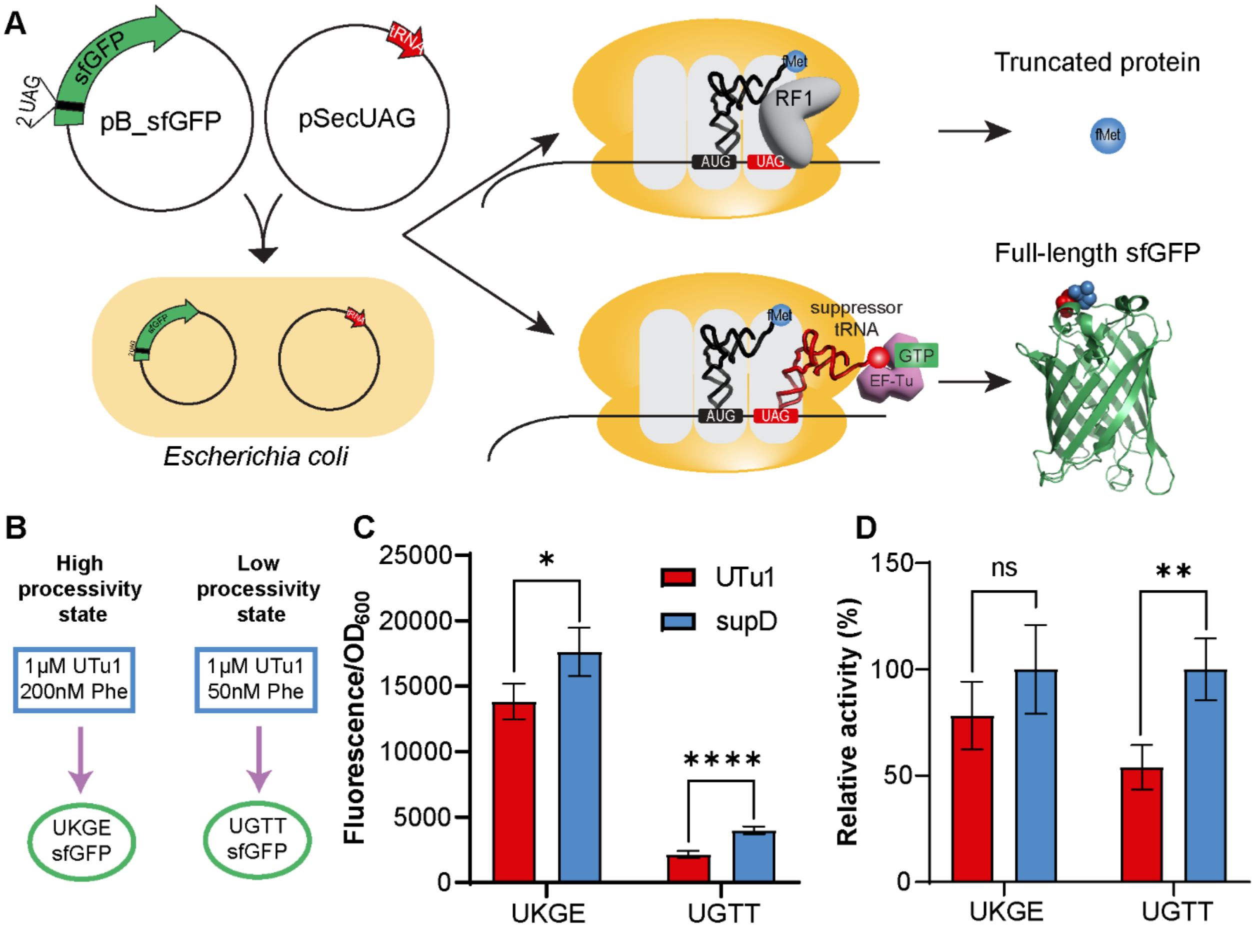

Supplementary Figure S7. Engineering an *in vivo* assay for a low processivity state. (**A**) Strategy for sfGFP readthrough assay which determines successful suppression of a UAG codon. (**B**) Schematic to demonstrate that sfGFP with the amino acids UKGE for positions 2-5 results in a high processivity state as observed with the addition of 200 nM Phe while amino acids UGTT results in a low processivity state as observed with the addition of 50 nM Phe. (**C**) Readthrough assay measured by fluorescence intensity over cell density of $tRNA^{UTu1}$ compared to $tRNA^{supD}$ in a high processivity (UKGE) and low processivity (UGTT) state. There is a significant decrease ($p<0.0001$) for $tRNA^{UTu1}$ compared to $tRNA^{supD}$ in the low processivity (UGTT) state. Error bars shown are the standard deviation from a minimum of four biological replicates. (**D**) Relative activity of sfGFP readthrough with $tRNA^{UTu1}$ compared to $tRNA^{supD}$ in a high processivity (UKGE) and low processivity (UGTT) state. There is a significant decrease ($p<0.01$) for $tRNA^{UTu1}$ in the low processivity (UGTT) state. Error bars are the standard deviation from a minimum of four biological replicates.

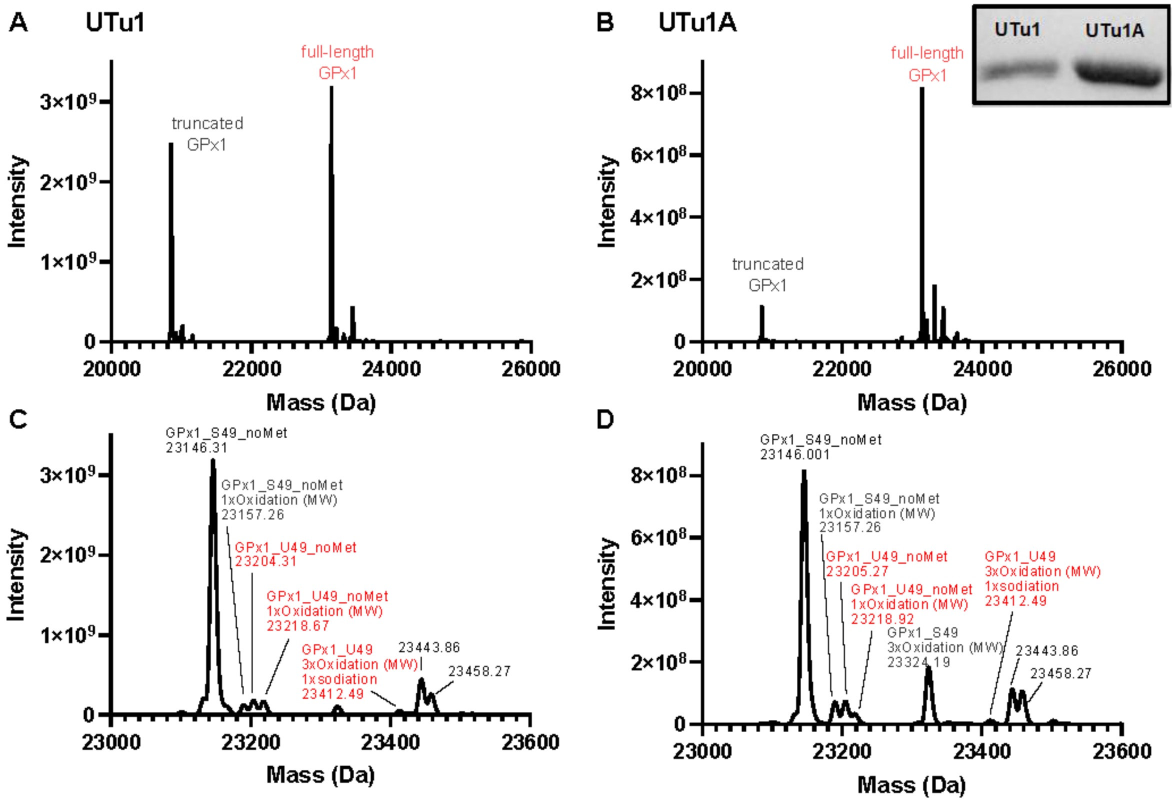

Supplementary Figure S8. Mass spectrometry analysis of GPx1. (**A**) Intact mass spectrum of GPx1 grown with tRNA^{UTu1} has a significant amount of truncated protein. (**B**) Intact mass spectrum of GPx1 grown with tRNA^{UTu1A} shows majority of sample as full-length protein. Gel inset of the purified proteins sent for analysis. (**C**) Zoom-in on the mass spectrum, focusing on full-length GPx1 grown with tRNA^{UTu1} highlighting the peaks containing Ser (in black) and Sec (in red). (**D**) Zoom-in on the mass spectrum, focusing on full-length GPx1 grown with tRNA^{UTu1A} highlighting the peaks containing Ser (in black) and Sec (in red).

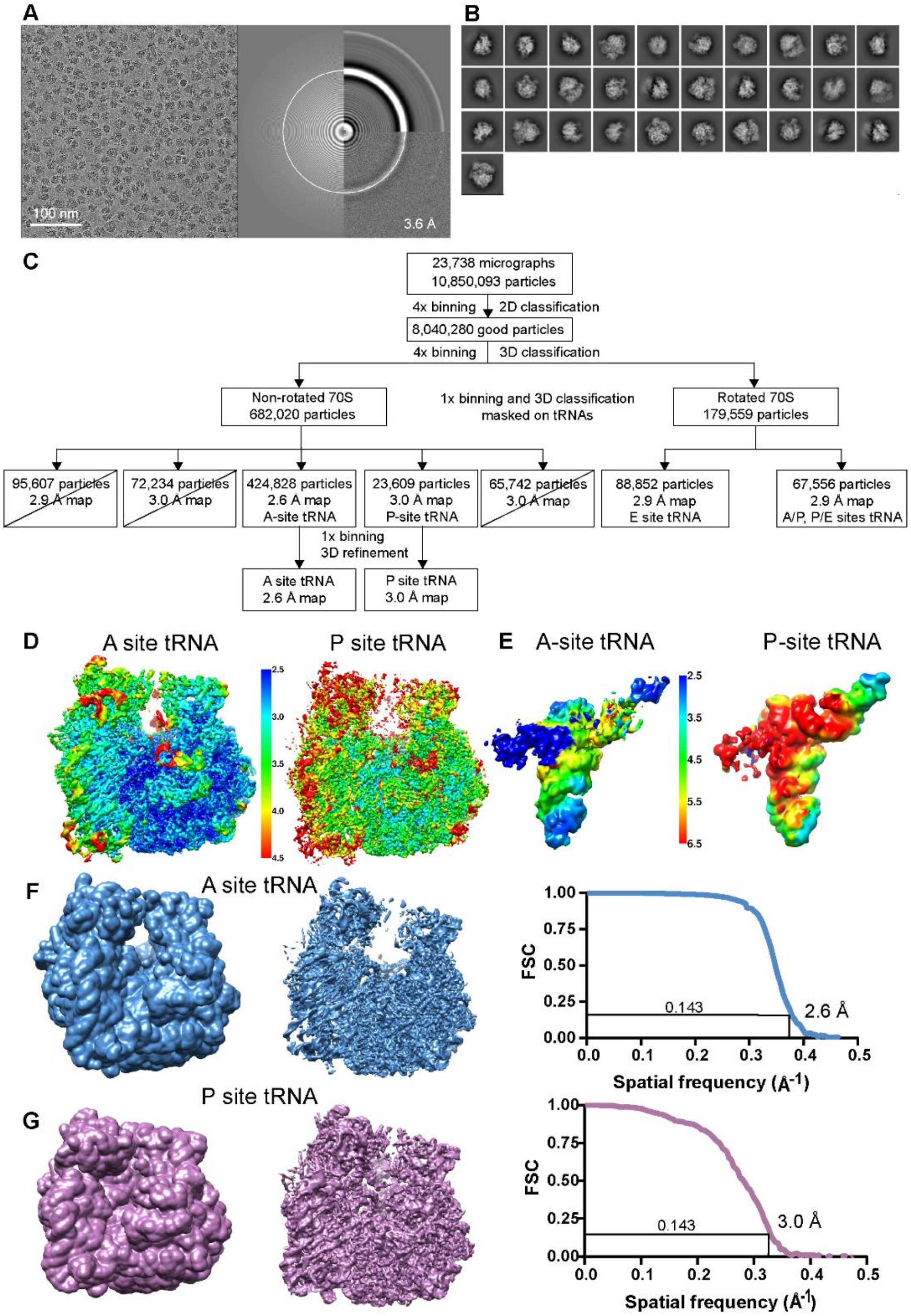

Supplementary Figure S9. Cryo-EM data processing of tRNA^{UTu1A} in complex with the ribosome. (**A**) Representative micrograph collected with a Titan Krios transmission electron microscope and corresponding power spectrum. (**B**) Representative 2D class averages from reference-free 2D classification. (**C**) Particle classification and structural refinement procedures used. (**D**) Local resolution estimation of the cryo-EM density map of the ribosome. The density map is displayed in surface representation and colored according to the local resolution (see color bar). (**E**) Local resolution estimation of the cryo-EM density map of tRNA^{UTu1A} in the A and P site of the ribosome. The density map is displayed in surface representation and colored according to the local resolution (see color bar). (**F**) The mask used for resolution estimation of the A site tRNA^{UTu1A} (left) including all components of the reconstruction. FSC curve for cryo-EM reconstruction (right). (**G**) The mask used for resolution estimation of the P site tRNA^{UTu1A} (left) including all components of the reconstruction. FSC curve for cryo-EM reconstruction (right).

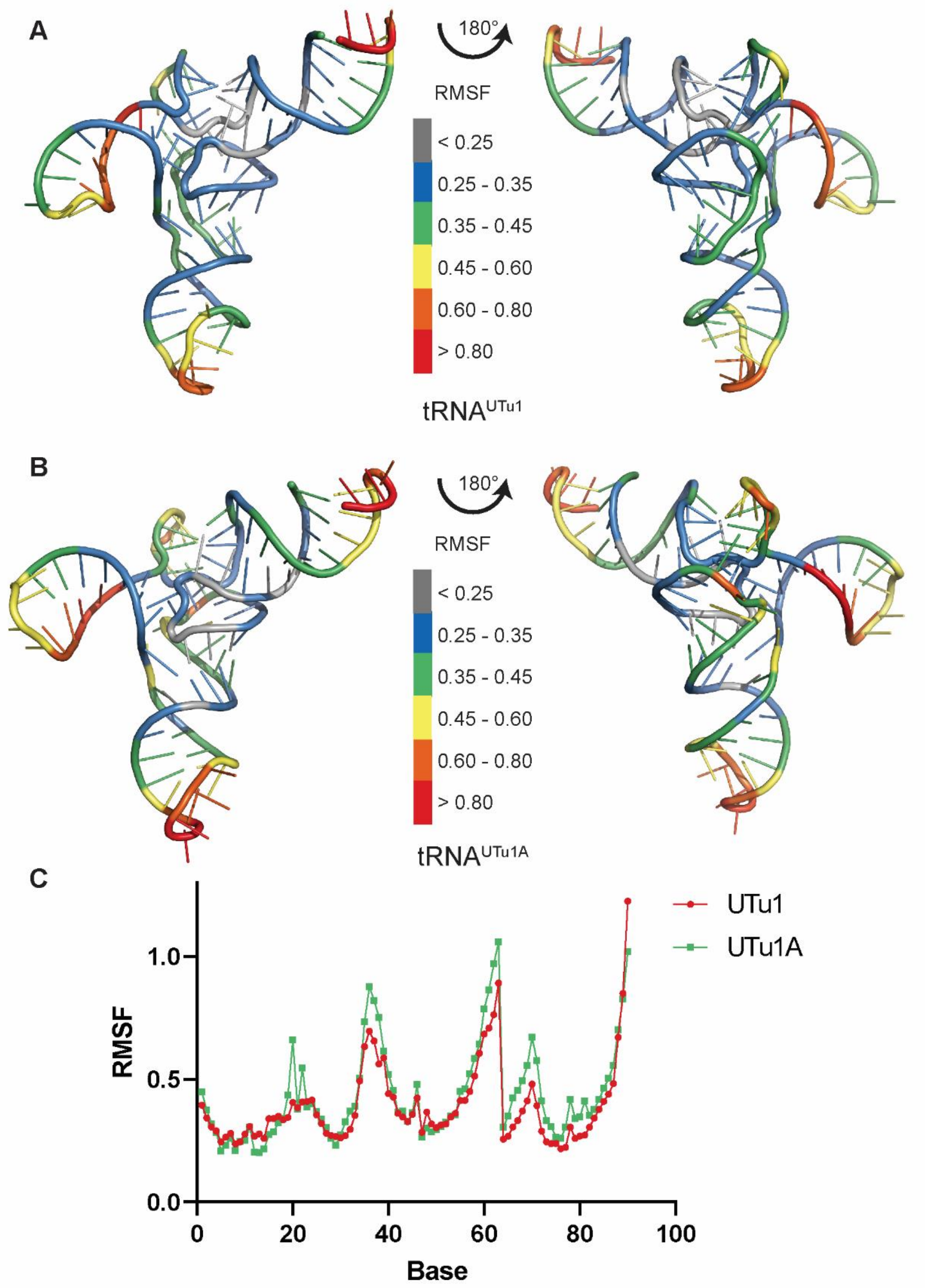

Supplementary Figure S10. Molecular dynamic simulations of tRNAs in solution. Representative average structure of (**A**) tRNA^{UTu1} and (**B**) tRNA^{UTu1A} colored according to the root mean squared fluctuation (RMSF) of that base in Å. (**C**) Trace of RMSF values for each base (using linear numbers). The RSMF values from the simulations suggest that tRNA^{UTu1A} is more flexible than tRNA^{UTu1}, particularly in the D-arm (base 19-22), variable arm (base 57-63), and the T-arm (base 66-73).

REFERENCES

1. Chung, C.Z., Krahn, N., Crnković, A. and Söll, D. (2021) Intein-based design expands diversity of selenocysteine reporters. *J Mol Biol*, 167199.
2. Mukai, T., Sevostyanova, A., Suzuki, T., Fu, X. and Söll, D. (2018) A facile method for producing selenocysteine-containing proteins. *Angew Chem Int Ed Engl*, **57**, 7215-7219.
3. Dorywalska, M., Blanchard, S.C., Gonzalez, R.L., Kim, H.D., Chu, S. and Puglisi, J.D. (2005) Site-specific labeling of the ribosome for single-molecule spectroscopy. *Nucleic Acids Res*, **33**, 182-189.
4. Marshall, R.A., Dorywalska, M. and Puglisi, J.D. (2008) Irreversible chemical steps control intersubunit dynamics during translation. *Proc Natl Acad Sci U S A*, **105**, 15364-15369.
5. Blanchard, S.C., Gonzalez, R.L., Kim, H.D., Chu, S. and Puglisi, J.D. (2004) tRNA selection and kinetic proofreading in translation. *Nat Struct Mol Biol*, **11**, 1008-1014.
6. Blanchard, S.C., Kim, H.D., Gonzalez, R.L., Jr., Puglisi, J.D. and Chu, S. (2004) tRNA dynamics on the ribosome during translation. *Proc Natl Acad Sci U S A*, **101**, 12893-12898.
7. Kitagawa, M., Ara, T., Arifuzzaman, M., Ioka-Nakamichi, T., Inamoto, E., Toyonaga, H. and Mori, H. (2005) Complete set of ORF clones of *Escherichia coli* ASKA library (a complete set of *E. coli* K-12 ORF archive): unique resources for biological research. *DNA Res*, **12**, 291-299.
8. Rio, D.C. (2013) Expression and purification of active recombinant T7 RNA polymerase from *E. coli*. *Cold Spring Harb Protoc*, **2013**.
9. Chen, J., Petrov, A., Tsai, A., O'Leary, S.E. and Puglisi, J.D. (2013) Coordinated conformational and compositional dynamics drive ribosome translocation. *Nat Struct Mol Biol*, **20**, 718-727.
10. Chen, J., Dalal, R.V., Petrov, A.N., Tsai, A., O'Leary, S.E., Chapin, K., Cheng, J., Ewan, M., Hsiung, P.L., Lundquist, P. *et al.* (2014) High-throughput platform for real-time monitoring of biological processes by multicolor single-molecule fluorescence. *Proc Natl Acad Sci U S A*, **111**, 664-669.
11. Zheng, S.Q., Palovcak, E., Armache, J.P., Verba, K.A., Cheng, Y. and Agard, D.A. (2017) MotionCor2: anisotropic correction of beam-induced motion for improved cryo-electron microscopy. *Nat Methods*, **14**, 331-332.
12. Zhang, K. (2016) Gctf: Real-time CTF determination and correction. *J Struct Biol*, **193**, 1-12.
13. Scheres, S.H. (2012) RELION: implementation of a Bayesian approach to cryo-EM structure determination. *J Struct Biol*, **180**, 519-530.
14. Kucukelbir, A., Sigworth, F.J. and Tagare, H.D. (2014) Quantifying the local resolution of cryo-EM density maps. *Nat Methods*, **11**, 63-65.
15. Pettersen, E.F., Goddard, T.D., Huang, C.C., Couch, G.S., Greenblatt, D.M., Meng, E.C. and Ferrin, T.E. (2004) UCSF Chimera--a visualization system for exploratory research and analysis. *J Comput Chem*, **25**, 1605-1612.
16. Emsley, P. and Cowtan, K. (2004) Coot: model-building tools for molecular graphics. *Acta Crystallogr D Biol Crystallogr*, **60**, 2126-2132.
17. Liebschner, D., Afonine, P.V., Baker, M.L., Bunkoczi, G., Chen, V.B., Croll, T.I., Hintze, B., Hung, L.W., Jain, S., McCoy, A.J. *et al.* (2019) Macromolecular structure determination using X-rays, neutrons and electrons: recent developments in Phenix. *Acta Crystallogr D Struct Biol*, **75**, 861-877.
18. Vagin, A.A., Steiner, R.A., Lebedev, A.A., Potterton, L., McNicholas, S., Long, F. and Murshudov, G.N. (2004) REFMAC5 dictionary: organization of prior chemical knowledge and guidelines for its use. *Acta Crystallogr D Biol Crystallogr*, **60**, 2184-2195.
19. Jorgensen, W.L., Chandrasekhar, J. and Madura, J.D. (1983) Comparison of simple potential functions for simulating liquid water. *J Chem Phys*, **79**, 926.

20. Huang, J. and MacKerell, A.D., Jr. (2013) CHARMM36 all-atom additive protein force field: validation based on comparison to NMR data. *J Comput Chem*, **34**, 2135-2145.
21. Lindahl, Abraham, Hess and Spoel, v.d. (2021). 2021 ed. Zenodo.
22. Lindahl, Abraham, Hess and Spoel, v.d. (2021). 2021 ed. Zenodo.
23. Hess, B. (2008) P-LINCS: A parallel linear constraint solver for molecular simulation. *J Chem Theory Comput*, **4**, 116-122.
